# Unmasking Lupus: Intracranial Hemorrhage as the Primary Clue in an Adolescent With Refractory Thrombocytopenia—A Case Report

**DOI:** 10.1155/crpe/7996356

**Published:** 2026-02-13

**Authors:** Bosaina Otour, Habiba Aboalela, Sulaima Taji, Razan Ismail, Ahmad AlHammada, Eman Omar

**Affiliations:** ^1^ Pediatric Emergency Department, Al Jalila Childrens Hospital, Dubai, UAE, aljalilahospital.com; ^2^ Pediatric Department, American Hospital, Dubai, UAE, amerikanhastanesi.org

## Abstract

Systemic lupus erythematosus (SLE) is a rare autoimmune disorder, rarely presenting with central nervous system (CNS) hemorrhage—especially cerebellar involvement. We report a previously healthy 17‐year‐old female presenting with severe thrombocytopenia and cerebellar hemorrhage, leading to a diagnosis of SLE. Recovery was dramatic and achieved by prompt multidisciplinary management, including neurosurgical intervention, immunosuppressive treatment, and intensive care. The case highlights the importance of considering SLE as a differential diagnosis of unexplained intracranial hemorrhage, particularly in adolescents presenting with cytopenia.

## 1. Introduction

Systemic lupus erythematosus (SLE) is a chronic autoimmune disease with multisystem involvement. Pediatric‐onset SLE (pSLE) constitutes 10%–20% of all SLE and follows a more aggressive course than adult‐onset disease [1]. It occurs with an incidence of 0.4–2.2 per 100,000 children and typically manifests with systemic symptoms such as fever, rash, arthritis, anemia, and thrombocytopenia [1–3]. Among these, hematologic abnormalities in the form of anemia and thrombocytopenia can be the presenting or sole initial clinical manifestations [2, 3].

Neuropsychiatric SLE (NPSLE) is present in 30%–70% of patients during the disease course but is rarely the initial presentation [4, 5]. Manifestations can vary from headache, seizures, cognitive impairment, and mood disorders, with central nervous system (CNS) involvement contributing significantly to disease burden. Intracranial hemorrhage is an uncommon complication, typically occurring as a consequence of SLE induced thrombocytopenia, vasculitis, or coagulopathy [6–9]. Though subdural, subarachnoid, and retinal bleeds have been described, to our knowledge, cerebellar hemorrhage as an initial manifestation of SLE has never been documented [6, 10]. This highlights the need to entertain autoimmune causes like SLE in patients presenting with unexplained CNS bleeding and hematological abnormalities [3, 10].

## 2. Case Presentation

A 17‐year‐old female patient presented to our emergency department with fatigue, pallor, and an unintentional 10 kg weight loss over one month. She developed headache, uncontrollable vomiting, blurred vision, and widespread ecchymosis and petechiae over 3 days prior to presentation. She was toxic and agitated on examination, with the aforementioned ecchymosis.

The first laboratory examinations showed severe anemia (Hb 6.4 g/dL) and profound thrombocytopenia (platelets 6 × 10^9^/L) in the context of a normal white blood cell count. Biochemical markers showed elevated lactate dehydrogenase and uric acid levels; the direct Coombs test was positive. The coagulation profile was normal, the peripheral smear had no schistocytes, and renal and liver function tests and urinalysis were normal (Table 1).

**Table 1:** Comprehensive laboratory investigations summary.

Investigation	Result	Normal range
Hemoglobin	6.4 g/dL	10.5–13.5 g/dL
White blood cells	6.9 × 10^3^/μL	5–19 × 10^3^/μL
Red blood cells	2.7 × 10^6^/μL	3.1–4.5 × 10^6^/μL
Platelets	6000/μL	150,000–450,000/μL
ESR	5 mm/1 h	< 13 mm/1 h
Urea	30 mg/dL	15.62–40.66 mg/dL
Creatinine	0.77 mg/dL	0.6–0.88 mg/dL
Peripheral smear	Bicytopenia and reactive picture	—
Direct Coombs test	Positive	Negative
DAT anti‐IgG Coombs	Positive 2+	Negative
DAT anti‐C3d	Positive 1+	Negative
PT	15.7 s	11.5–14.5 s
APTT	34 s	28.6–38.2 s
PT control	13.3 s	—
D‐dimer test	3.09 (high)	< 0.5 μg/mL FEU
APTT mix	Prolonged PT corrected by mixing studies with normal pool plasma: 14 s	—

Neuroimaging demonstrated multiple cerebellar hemorrhages with mass effect resulting in compression of the fourth ventricle, brainstem compression, mild tonsillar herniation, and hydrocephalus—a life‐threatening but rare presentation (Figure 1).

Figure 1(a, b) Neuroimaging findings. (a) Axial CT head showing the highlighted abnormal area (blue arrow). (b) Sagittal CT head demonstrating the corresponding lesion (blue arrow).

**Figure:**
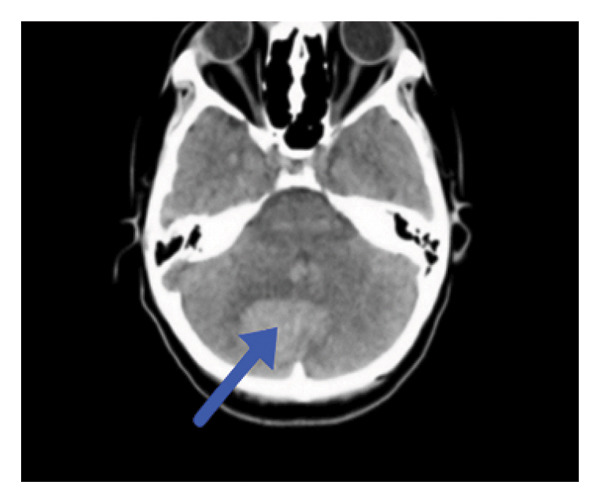
(a)

**Figure:**
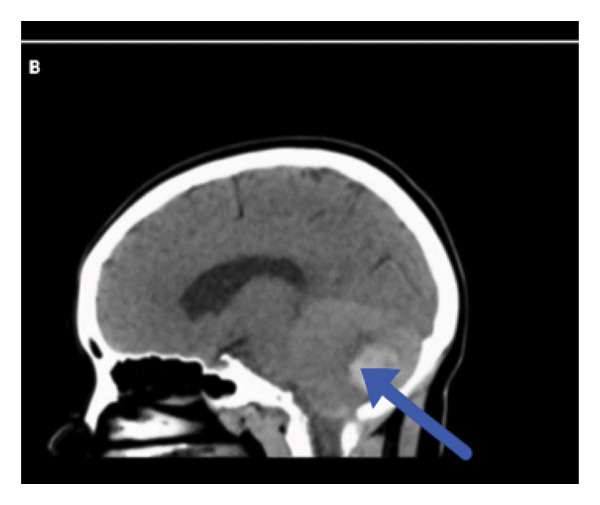
(b)

The initial management included stabilization of the patient on an emergency basis. Platelet transfusion and packed red blood cells were transfused, and adjuncts such as recombinant factor VIIa and tranexamic acid were used to limit bleeding. An external ventricular drain (EVD) was urgently inserted to decompress elevated intracranial pressure,

Balancing life‐saving treatment with the hemorrhagic risk posed by severe thrombocytopenia was a major clinical challenge. High‐dose intravenous corticosteroids, intravenous immunoglobulin (IVIG), and romiplostim were initiated to promote platelet recovery in refractory immune thrombocytopenia. Despite these measures, platelet counts remained critically low, raising suspicion of an underlying autoimmune process. Further immunologic evaluation revealed positive antinuclear antibody (ANA) and anti‐double‐stranded DNA titers, hypocomplementemia, and negative antiphospholipid antibodies, confirming a diagnosis of SLE (Table 2).

**Table 2:** Immunological workup results.

Investigation	Result	Normal range
Complement—C4	0.09 (low) g/L	0.10–0.40 g/L
Complement—C3	0.83 (low) g/L	0.90–1.80 g/L
ENA‐profile	Negative	—
Antinuclear antibody (ANA)	Positive 1/100; homogenous	< 1/100;
Anti‐dsDNA	737.31 IU/mL (positive)	< 100 IU/m
Anti‐cardiolipin IgG	13.3 CU (negative)	< 20 CU
Anti‐cardiolipin IgM	2.4 CU (negative)	< 20 CU
Anti‐ß2‐glycoprotein 1 IgG	9.0 CU (negative)	< 20
Anti‐ß2‐glycoprotein 1 IgM	< 1.1 CU (negative)	< 20

**Figure 2:**
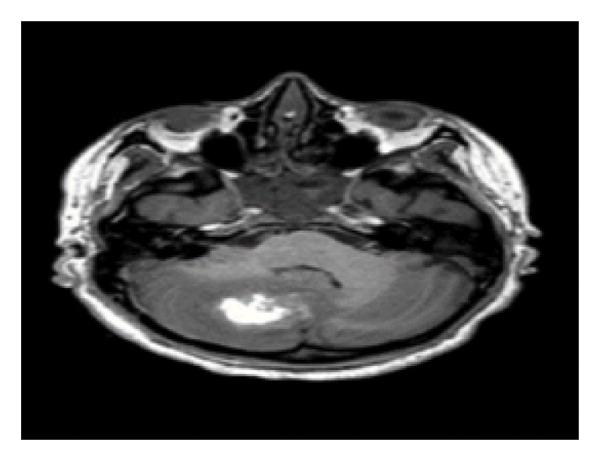
Axial MRI brain (T2‐weighted/FLAIR) showing a cerebellar hematoma.

**Figure 4:**
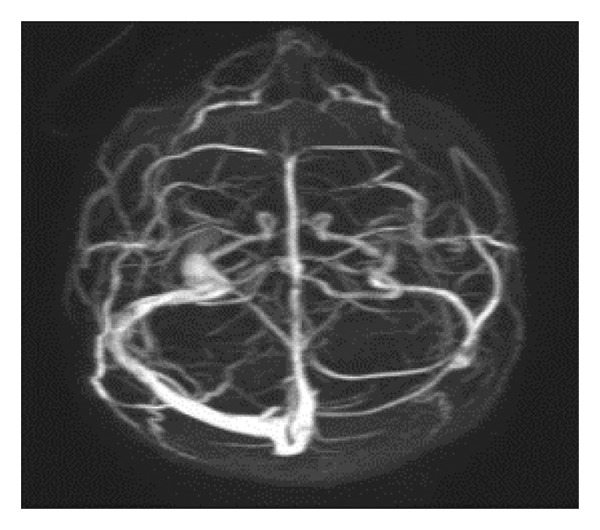
Magnetic resonance venography (MRV) demonstrating patent venous sinuses without thrombosis or abnormalities.

**Figure 5:**
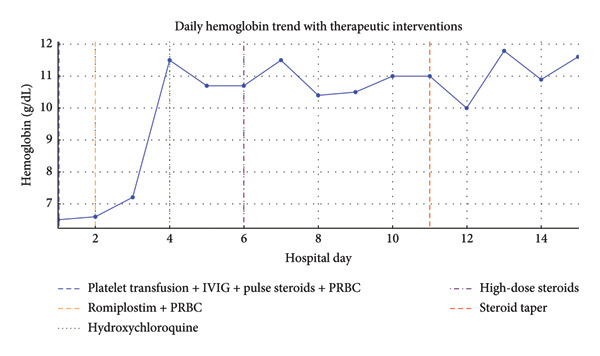
Hemoglobin trend with therapeutic interventions. Hemoglobin levels were low on admission but stabilized after early PRBC transfusions. Vertical dashed lines indicate the timing of transfusions and subsequent immunomodulatory treatments.

**Figure 6:**
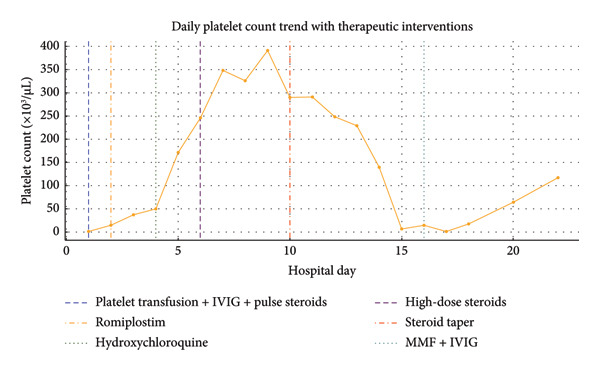
Daily platelet count trend with therapeutic interventions. Platelet counts during hospitalization showing severe thrombocytopenia on admission with fluctuations corresponding to treatment. Vertical dashed lines indicate initiation of platelet transfusion with IVIG and pulse steroids, romiplostim, hydroxychloroquine, high‐dose corticosteroids, steroid taper, and later MMF with IVIG.

MRI, MRA, and MRV studies showed no evidence of CNS vasculitis (Figures 2, 3, 4) supporting immune‐mediated thrombocytopenia as the most likely cause of the intracranial hemorrhages. Following the SLE diagnosis, disease‐modifying therapy was commenced with hydroxychloroquine, followed by mycophenolate mofetil to achieve immunosuppression and prednisone for control of persistent hematologic symptoms (Figures 5 and 6).

Figure 3Magnetic resonance angiography (MRA) of the brain showing normal cerebral arteries with no evidence of aneurysm, vasculitis, or vascular malformations. (a) Axial view. (b) Sagittal view.

**Figure:**
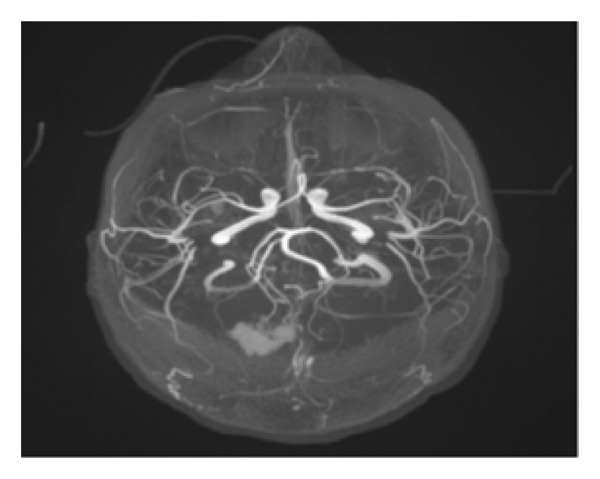
(a)

**Figure:**
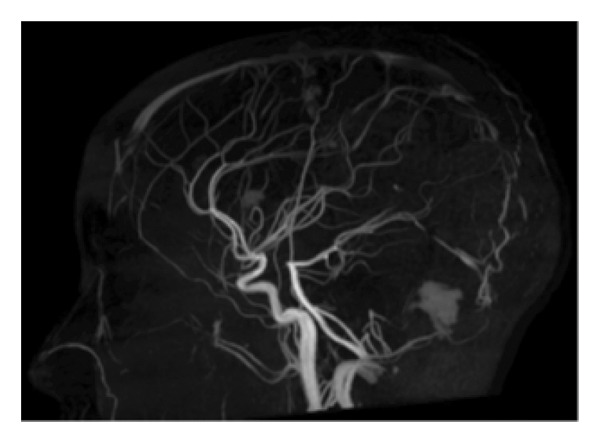
(b)

The patient’s course was complicated by fluid overload, opportunistic infections, and retinal vasculitis resulting in loss of vision in the left eye. Nevertheless, with meticulous intensive care and maintenance of immunosuppressive therapy, gradual neurological improvement was achieved.

## 3. Discussion

Cerebellar hemorrhage as the first sign of pediatric SLE is extremely rare and often linked to isolated immune‐mediated thrombocytopenia; both are infrequently reported together in the literature [2, 9, 10]. Isolated thrombocytopenia is recognized as an early marker of SLE and independently increases the risk of intracranial hemorrhage, with an odds ratio around 3.7 [3–5]. Our patient’s lack of antiphospholipid antibodies and the absence of vasculitis on imaging support immune thrombocytopenia as the main cause of her bleeding [3, 5]. Thrombocytopenia may precede other lupus symptoms and occasionally be the only initial clinical clue.

The combination of profound thrombocytopenia and intracranial hemorrhage requires exclusion of thrombotic thrombocytopenic purpura (TTP), hematologic malignancy, and infection‐associated thrombocytopenia. TTP was unlikely due to the absence of schistocytes. Malignancy was excluded by normal peripheral smear without blasts and absence of organomegaly. Infectious causes were ruled out by a negative workup and serology consistent with SLE.

Though intracranial hemorrhage in SLE is rare, it has high morbidity, and hence early diagnosis and management are essential [4]. Emergency management was complicated by the fact that insertion of an EVD was high risk for bleeding in the setting of severe thrombocytopenia [4, 9]. Platelet transfusions were administered to the patient in an effort to reduce this risk, but these are of potentially limited value in immune‐mediated thrombocytopenia [8, 9, 11]. Therefore, adjunctive hemostatic agents, including recombinant factor VIIa and tranexamic acid, were administered to achieve surgical hemostasis [8]. High‐dose corticosteroids were administered as first‐line immunosuppressive therapy (in accordance with American College of Rheumatology/European Alliance of Associations for Rheumatology [ACR/EULAR] recommendations, in addition to IVIG) [11].

Romiplostim, a thrombopoietin receptor agonist, was initiated in this case due to persistent severe thrombocytopenia despite high‐dose corticosteroids and IVIG. Although primarily approved for immune thrombocytopenia, emerging evidence supports its use in refractory SLE‐associated thrombocytopenia, particularly when rapid platelet recovery is required to prevent life‐threatening bleeding [8, 10, 11]. By stimulating megakaryocyte activity and platelet production, romiplostim served as an effective adjunct while disease‐modifying immunosuppressive therapy took effect, contributing to gradual hematologic stabilization in our patient.

After stabilization, the patient was switched to long‐term maintenance therapy with hydroxychloroquine and mycophenolate mofetil, as is the practice in severe pSLE [11].

This case broadens the clinical spectrum of pSLE and illustrates that autoimmune causes need to be considered in teenagers with unexplained CNS hemorrhage and cytopenia [5, 10]. It illustrates the importance of early immunologic evaluation and urgent multidisciplinary management to optimize outcomes in such high‐risk cases.

## 4. Conclusion

Cerebellar hemorrhage as a first manifestation of pediatric SLE is extremely rare but can be life‐threatening, especially in the context of severe thrombocytopenia. The case highlights the extreme importance of early immunological studies and the initiation of appropriate immunosuppressive therapy. The use of corticosteroids, IVIG, romiplostim, and MMF together was successful in facilitating recovery.

## Consent

All the patients allowed personal data processing, and informed consent was obtained from all individual participants included in the study.

## Conflicts of Interest

The authors declare no conflicts of interest.

## Funding

No funding was received for this study.

## Data Availability

The data that support the findings of this study are available from the corresponding author upon reasonable request.
